# A genome-wide RNAi screen identifies the SMC5/6 complex as a non-redundant regulator of a Topo2a-dependent G2 arrest

**DOI:** 10.1093/nar/gky1295

**Published:** 2018-12-24

**Authors:** Katharina Deiss, Nicola Lockwood, Michael Howell, Hendrika Alida Segeren, Rebecca E Saunders, Probir Chakravarty, Tanya N Soliman, Silvia Martini, Nuno Rocha, Robert Semple, Lykourgos-Panagiotis Zalmas, Peter J Parker

**Affiliations:** 1 Protein Phosphorylation Laboratory, The Francis Crick Institute, 1 Midland Road, London NW1 1AT, UK; 2 High Throughput Screening, The Francis Crick Institute, 1 Midland Road, London NW1 1AT, UK; 3 Bioinformatics, The Francis Crick Institute, 1 Midland Road, London NW1 1AT, UK; 4 The University of Cambridge Metabolic Research Laboratories, Wellcome Trust-MRC Institute of Metabolic Science, Cambridge CB2 0QQ, UK; 5 The National Institute for Health Research Cambridge Biomedical Research Centre, Cambridge CB2 0QQ, UK; 6 Translational Cancer Therapeutics, The Francis Crick Institute, 1 Midland Road, London NW1 1AT, UK; 7 School of Cancer and Pharmaceutical Sciences King's College London, New Hunt's House, Guy's Campus, London SE1 1UL, UK

## Abstract

The Topo2a-dependent arrest is associated with faithful segregation of sister chromatids and has been identified as dysfunctional in numerous tumour cell lines. This genome-protecting pathway is poorly understood and its characterization is of significant interest, potentially offering interventional opportunities in relation to synthetic lethal behaviours in arrest-defective tumours. Using the catalytic Topo2a inhibitor ICRF193, we have performed a genome-wide siRNA screen in arrest-competent, non-transformed cells, to identify genes essential for this arrest mechanism. In addition, we have counter-screened several DNA-damaging agents and demonstrate that the Topo2a-dependent arrest is genetically distinct from DNA damage checkpoints. We identify the components of the SMC5/6 complex, including the activity of the E3 SUMO ligase NSE2, as non-redundant players that control the timing of the Topo2a-dependent arrest in G2. We have independently verified the NSE2 requirement in fibroblasts from patients with germline mutations that cause severely reduced levels of NSE2. Through imaging Topo2a-dependent G2 arrested cells, an increased interaction between Topo2a and NSE2 is observed at PML bodies, which are known SUMOylation hotspots. We demonstrate that Topo2a is SUMOylated in an ICRF193-dependent manner by NSE2 at a novel non-canonical site (K1520) and that K1520 sumoylation is required for chromosome segregation but not the G2 arrest.

## INTRODUCTION

Cell cycle checkpoints delay progression if aberrant or incomplete cell cycle events such as damaged or incompletely replicated DNA are detected, thereby providing time for the cell to resolve the problem (1,2). These are critical for the precise inheritance of the parental genome, with defective checkpoints being a hallmark of cancer cells contributing to genetic instability and malignant transformation (2–4). An intriguing but poorly understood cell cycle arrest is the Topo2a-dependent arrest in G2, which has often been referred to as a decatenation checkpoint (5). Topo2a itself is known to be required for this G2 arrest (6,7) and although it is established that there is increased catenation consequent to inhibition (8) it is not known whether catenation as such is being sensed by cells. Moreover, this arrest has not formally been identified as a *bona fide* cell cycle checkpoint involving sensors, transducers and effectors, so we will refer to it here as a Topo2a-dependent G2 arrest.

The Topo2a-dependent G2 arrest can be studied using the tool compound ICRF193, a catalytic Topo2 inhibitor that prevents the enzyme release from DNA after DNA double-strand break religation (9). By contrast, the so-called Topo2 poisons such as etoposide stabilize the covalent enzyme–DNA complex prior to the religation step in the catalytic cycle and are hence associated with double strand breaks, typically triggering a DNA damage response. The dysfunction of the Topo2a-dependent arrest in a plethora of transformed cell lines (8,10–12) questions the selective pressures exerted on the unknown mechanisms that generate this arrest and also highlights the clinical potential of the downstream failsafe pathway(s) as targets for chemotherapy. These both prescribe a more detailed understanding of the genes involved in this G2 arrest and their possible dysfunction in cancer. We therefore designed a genome-wide siRNA screen to identify genes that are necessary to arrest cell cycle progression under conditions of compromised Topo2a activity using the catalytic inhibitor ICRF193. Previous studies are limited because they predominantly identified putative players using transformed cell lines (5,6,13–16). Given the various cell cycle abnormalities associated with cancer cell lines, the present study was designed to characterize the non-redundant requirements for the Topo2a-dependent G2 arrest in non-transformed cells.

Here, we unequivocally show that the Topo2a-dependent arrest is genetically distinguishable from the DNA damage checkpoint using a multistep screening approach. The validity of the screen is evident from the identification of Topo2a itself, a known requirement for this arrest, and additionally five of six subunits of the SMC5/6 complex as strong hits. Subsequent studies, including the use of patient-derived cells with severely reduced levels of NSE2, implicated the SMC5/6 complex and its NSE2 SUMO E3 ligase subunit in this arrest and resolution pathway. Mechanistically, we have determined that upon ICRF193 engagement of a G2 arrest there is an increased interaction of Topo2a with the SMC5/6 subunit NSE2 at PML bodies. We also reveal Topo2a as a previously unrecognized target of the NSE2 E3 SUMO ligase and go on to show that SUMOylation of Topo2a at a novel site, K1520, is required for chromosome segregation, but not the Topo2a-dependent G2 arrest. The evidence points to a G2 arrest mechanism distinct from DNA damage checkpoint controls that engages genes previously characterized in other contexts, but for which their action in determining G2 progression following Topo2a inhibition has hitherto been unrecognized.

## MATERIALS AND METHODS

### Reagents

For a full list of reagents please see Supplementary Table S1.

### Cell lines

All cell lines were maintained at 37°C. For cell line authentication, cell lines were mycoplasma screened, speciated and STR profiled. The STR profile was cross referenced back to any available published profile for the line in question. If there was no published profile available, it was checked against the Cell Services STP database of the Francis Crick Institute to ensure that it gives a unique profile. Please see Supplementary Table S2 for further information.

For cDNA transfection of Doxycycline-inducible U2OS cell lines X-tremeGENE 9 (Sigma) was used according the manufacturer's instructions and selection was carried out at a concentration of 0.2 mg/ml Hygromycin (Invitrogen). To induce Topo2a-GFP expression cells were cultured with Doxycycline (100 ng/ml) for 24 h prior to assay for asynchronous populations or with the first thymidine block for synchronous/synchronised populations. Stable RPE1 cell lines were generated using Lipofectamine LTX (Invitrogen) and stable H2170 cells were generated using Fugene according to the manufacturer's instructions and selection was carried out at a concentration of 0.8 mg/ml G418 (Sigma).

### RNAi screen and G2 arrest assay

RPE1 cells were reverse-transfected with the human siRNA Smartpool siGenome library from Dharmacon in black 384 well plates in triplicate using Lullaby transfection reagent [OZ Biosciences, 900cells/well, 0.1μL Lullaby/well, 19nM siRNA and a number of Xrd-384 liquid dispensers (FluidX)]. After 55 h, cells were treated for 18 h (for drug concentrations see table below). This treatment duration was chosen as it was long enough to allow sufficient cells to go through the cell cycle but short enough to not allow mitotic slippage. Cells were subsequently fixed with Methanol/Acetic Acid (95/5) overnight at −20°C. Cells were rehydrated in PBS, blocked in 2.5% BSA in PBS and stained using Cy5-conjugated MPM2 (Millipore) and DAPI (Merck). Images were acquired and analyzed using both an ArrayScan VTi-automated microscope (Cellomics; image analysis using the Target activation Bioapplication) and an Acumen Explorer eX3 laser scanning microplate cytometer (TTPLabtech data analysis using the Cellista analysis package). For each well the mitotic index (MI) was determined [(Number of MPM2 positive cells/total number of cells) × 100] and for the primary screen a median *z*-score was derived using plate normalization [(well value − plate median)/plate Median Absolute Deviation]. For the validation screens *z*-score normalization is not appropriate as only a subset of the population is being analysed, which are extreme effectors from the primary screen. Therefore, results are expressed as a percentage of control, derived as [(well value/plate mean Risc-free well) × 100]. For clarity, the logged normalized MI are plotted. Throughout the paper, each data point from any screen represents the median of *n* = 3 technical replicates of an individual siRNA pool. When the arrest behaviour was assessed independent of the screen at least six technical replicates were performed, the experiment was repeated three times and the percentage of mitotic cells was determined through normalization to 1 μM Nocodazole only treatment. The same protocol was used for all screening rounds and subsequent arrest assays. The following criteria were used for hit selection:

**Table:** 

Screen	Normalization	Condition	Cut-off	Image acquisition
Primary screen	*z*-score	3 μM ICRF193 + 1 μM Nocodazole	MI^ICRF^ > 2.5 MI^ICRF^ > 2.5	ArrayScan Acumen
			Cell number > –1.5	Acumen
	POC*	3 μM ICRF193 + 1 μM Nocodazole	MI^ICRF^ > 350 MI^ICRF^ > 200	ArrayScan Acumen
		10 μM Bleomycin +1 μM Nocodazole	MI^Bleo^ < 200 MI^Bleo^ < 200	ArrayScan Acumen
		No drug	MI^nodrug^ < 150 MI^nodrug^ < 200	ArrayScan Acumen
Deconvoluted screen	POC	3 μM ICRF193 + 1 μM Nocodazole	MI^ICRF^ > 150	ArrayScan
Extended counter-screen	POC	3 μM ICRF193 + 1 μM Nocodazole	MI^ICRF^ > 350	ArrayScan
		10 μM Bleomycin + 1 μM Nocodazole	MI^ICRF^ > 2*MI^Bleo^	ArrayScan
		1 μM Etoposide + 1 μM Nocodazole	MI^ICRF^ > 2*MI^Etop^	ArrayScan
		10 GY γ-irradiation +1 μM Nocodazole	MI^ICRF^ > 2*MI^γ-irrad^	ArrayScan
		No drug	MI^ICRF^ > 2*MI^nodrug^	ArrayScan

*POC: percentage of control

### Cell synchronization

Cells were synchronized in early S phase through performing a double thymidine block. Cells were cultured for 16 h in growth medium supplemented with 2.5 mM thymidine 6 h after reverse transfection of the indicated siRNAs. Subsequently, they were washed and released into growth medium for 6 h before an additional 16 h incubation with 2.5 mM thymidine. Cells were then released into growth medium for 5 or 10 h (as indicated) before additional drug treatments, allowing specific analysis of cells treated in G2.

### Co-immunoprecipitation and Immunoblotting

Cells were lysed by sonication in ice-cold RIPA-buffer (1% Triton X, 1% sodium deoxycholate, 0.1% SDS, 150 mM NaCl, 1 mM EDTA, 50 mM TRIS pH 7.4) supplemented with Complete EDTA-free Protease Inhibitor Cocktail (Roche), 1 mM PMSF and where indicated 25 mM iodoacetamide. Additionally, where indicated lysates were treated with 0.02 U/ml Benzonase Nuclease supplemented with 1 mM MgCl_2_ for 1 h at 4°C. After centrifugation (14 000*g*, 4°C, 10min), the supernatant was incubated with Topo2a-antibodies (Millipore) or SMC6-antibodies (Abcam) bound to Dynabeads Protein G (Invitrogen) or with HA-agarose beads (Sigma) for 2 h at 4°C. Immunoprecipitated proteins were washed using RIPA buffer, eluted using LDS-sample buffer (Invitrogen) for 5 min at 95°C, separated by SDS-PAGE and transferred to PVDF membranes. Membranes were blocked overnight in either 5% fat-free milk dissolved in PBS + 0.1%Tween20 (PBST) for the following antibodies: mouse monoclonal anti-HA (Covance), rabbit polyclonal anti-SMC6 (Abcam), mouse monoclonal anti-Topo2a (Millipore), rabbit monoclonal anti-Topo2a (Abcam), rabbit polyclonal anti-Topo2a (Topogen), mouse anti-alpha tubulin (Sigma), or with 2.5% BSA in PBS for probing with rabbit polyclonal anti-SUMO2/3 (Enzo) or with Streptavidin-HRP (Sigma). Antibodies were subsequently detected using HRP-conjugated secondary anti-rabbit and anti-mouse antibodies (GE Healthcare) and Luminata HRP substrate (Millipore). A representative image of at least three experiments is shown.

### Flow cytometry

Cells were stained with either Aqua or Blue LIVE/DEAD™ Fixable Dead Cell Stains (ThermoFisher Scientific) according to manufacturer's instructions, prior to fixation in ice-cold 70% ethanol. Subsequently cells were permeabilized with 0.1% Triton X and stained at RT in the dark for 1 h with either anti-GFP (Abcam) followed by anti-rabbit Alexa Fluor488 (Invitrogen) and anti-MPM2-Cy5 (Millipore) or anti-MPM2-Cy5 (Millipore) alone. Samples were then stained with 50 mg/ml propidium iodide (PI) with 100 μg/ml RNaseA at RT in the dark for 30 min and left at 4°C overnight. Flow cytometry was conducted using an LSR or Fortessa flow cytometer and acquisition software FACSDiva (BD bioscience). FACS analysis was performed using the FlowJo software and gating for Aqua or Blue negative (live) cells, single cells with PI staining and, where required, GFP gating was applied. Cell cycle distributions were calculated using the integrated Watson-Pragmatic algorithm.

### RNA preparation and real-time PCR

RPE1 cells were transfected with siRNAs targeting SMC6 or non-targeting controls using Lullaby transfection reagent. 56 h later, RNA was isolated from RPE1 cells using the RNeasy^®^-Kit (Qiagen). Reverse transcription and Real-time PCR was performed using QuantiTect SYBR Green RT-PCR Kit and the Applied Biosystems 7500 Fast System Real-time cycler. Primers are described in Supplementary Table S1. We analysed data using the 2^−ΔΔCT^-method with GAPDH as internal standard. Gene expression was normalized to gene expression in the control siRNA-transfected samples. Mean and S.D. of three experiments were determined.

### Immunofluorescence imaging

Cells were grown on 13 mm glass coverslips and pre-extracted with 0.1% Triton X in PBS for 30 s (all experiments except for IF analysis of segregation errors using anti-PICH antibodies), fixed using 4% PFA in PBS for 10 min, re-permeabilized using 0.1% Triton X in PBS for 5 min and blocked in 2.5% BSA in PBS for 1 h. The following primary antibodies were used: rabbit polyclonal anti-GFP (Abcam), rabbit polyclonal anti-PICH, (Novus), rabbit monoclonal anti-PML-Alexa Fluor555 (Abcam), rabbit polyclonal anti-SMC6 (Abcam), rabbit polyclonal anti-SUMO2/3 (Enzo), mouse monoclonal anti-Topoisomerase2a (Millipore), mouse monoclonal anti-PML (Abcam). Primary antibodies were detected with Alexa Fluor488, 555 or 647 conjugated secondary antibodies (Life technologies). All coverslips were mounted using ProLong Gold Diamond (Invitrogen). Images were taken using an inverted or upright laser scanning confocal microscope (Carl Zeiss LSM 780 or 880) equipped with a 40× or 63× Plan-APOCHROMAT DIC oil-immersion objective.

### PLA

RPE1 cells were grown on 8 well chambered slides (Falcon). Cells were reverse transfected and drug treated as for the RNAi screen and fixed as for immunofluorescent imaging. Proximity ligation assays were conducted using a kit (Sigma) as per manufacturer's instructions.

### IF image analysis

All immunofluorescence images were quantified using a custom-built script and the commercial software package MATLAB (MATLAB 2016a, MathWorks). The images were first segmented through the DAPI channel to subsequently enable nuclear foci detection through a percentile based intensity filter. Nuclear area was used when quantifying the number of foci to prevent bias as a result of drug treatment altering nuclear size. For colocalization quantification, the identified foci were compared across the channels and the percentage that colocalized were identified. At least 10 images were analysed and the mean and S.E.M. of at least three experiments was quantified.

### Chromosome spreads

For measurement of catenation, RPE1 cells were transfected with siRNAs targeting SMC6 or non-targeting controls using Lullaby transfection reagent. 48 h later, cells were retransfected with the same siRNAs together with siSgo1 for 24 h, followed by 12 h treatment with 1 μM Nocodazole to collapse the mitotic spindle. Cells were collected by shaking off the mitotic cells and resuspending them in a hypotonic solution of 75 mM KCl and incubation at 37°C for 30 min to expand the cells. Cells were then resuspended in 3:1 methanol:acetic acid and fixed overnight at –20°C. Cells were then washed using 3:1 methanol:acetic acid and spread onto clear slides by dropping from a height of 2 m. For assays where Topo2a was reintroduced, recombinant Topo2a (1 U/ml, TopoGen) was added in the hypotonic step where the cell membrane becomes hyperpermeable. The hypotonic buffer used here contained 5 mM TRIS, pH 8.0, 75 mM KCl, 10 mM MgCl_2_, 2 mM ATP, 0.5 mM dithiothreitol. Catenation was determined as the percentage of catenated sister chromatids per cell (*n* = 15) and the mean and S.E.M. of three experiments was calculated. Representative images are shown.

### *In vitro* SUMOylation

SUMOylation assays were performed using a SUMOylation kit (Enzo) according to the manufacturer's instructions. Where indicated, 15 ng of human purified Topo2a (Topogen) or 100 ng of purified biotinylated peptides and 100 ng of NSE2-GST (Abnova), PIAS1-GST (Enzo) or PIAS4-GST (Abnova) were added and the reaction was incubated for 45 min at 37°C.

### SMC5/6 expression analysis in LUSC patients

RSEM normalized gene expression RNASEQ TCGA data was downloaded from the FireHose website [https://gdac.broadinstitute.org/]. Gene expression values were ranked and the upper and lower pentile samples were used to draw survival plots using the survival package (version 2.40.1) in R (version 3.3.3). Statistical significance was tested using the log-rank test.

### Statistical analysis

For experiments where the data include more than two conditions, a two-way analysis of variance (ANOVA) using multiple comparisons was used, in all other cases an unpaired *t*-test was used for analysis as indicated in figure legends. Statistical details of individual experiments are given in the figure legends or Materials and Methods section. Prism software (Graphpad) was used for all calculations. For analysis of statistical significance of survival curves a log-rank test was used. The level of statistical significance is represented as follows: not significant (ns) = *P* > 0.05, *P ≤ 0.05, **P ≤ 0.01, ***P ≤ 0.001 and ****P ≤ 0.0001.

## RESULTS

### A genome-wide RNAi screen shows that the Topo2a-dependent G2 arrest is mechanistically distinct from the DNA damage checkpoint

**Figure 1.**
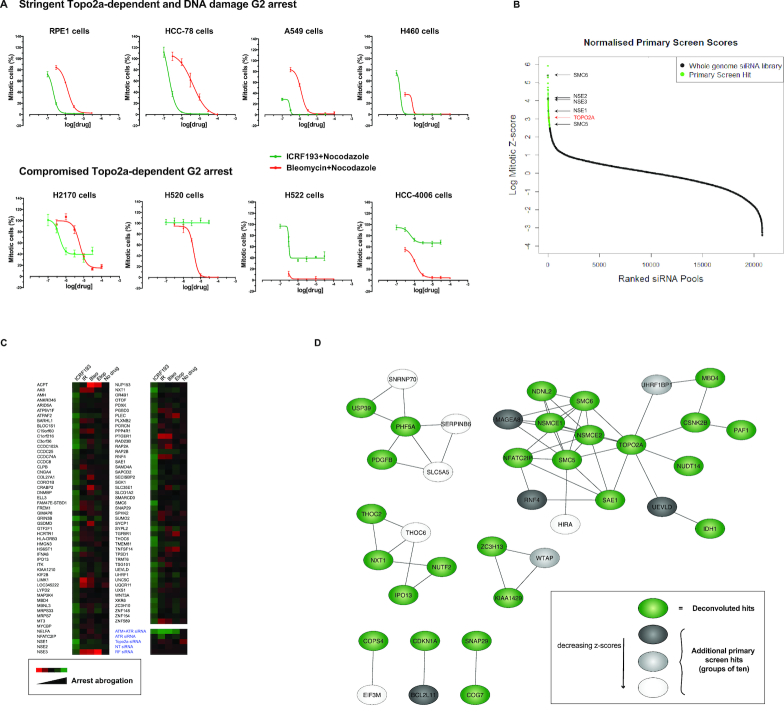
A genome-wide RNAi screen shows that the Topo2a-dependent G2 arrest is mechanistically distinct from the DNA damage checkpoint and requires the SMC5/6 complex. (**A**) Graphs showing the responses of the indicated asynchronous cell lines in response to increasing concentrations of ICRF193 or Bleomycin for 24 h. The bypass of arrest population was blocked in mitosis by inclusion of 1 μM Nocodazole and identified by staining for MPM2, yielding a mitotic index (MI) score through automated IF analysis. Data are represented as mean ± S.D. of a representative experiment with eight technical replicates, where the percentage of mitotic cells is normalized to the Nocodazole only treated control. *n* = 2–3. (**B**) Distribution of the normalized MI of the genome-wide siRNA library. This data is also part of Supplementary Figure S1E. (**C**) Heat-map of the arrest abrogation effects of the ICRF193-selected hits in an extended counter-screen with multiple distinct chromatin stresses (IR, ionising radiation; Bleo, bleomycin; Etop, etoposide). (**D**) The predicted protein-protein interactions for the 48 deconvoluted hits were compiled from the STRING database (green). To this we added additional hits from the primary screen, which were sequentially added in groups of ten based on their ranked *z*-scores (grey > light grey > white). An additional 30 genes were added before reaching the enrichment score threshold for background noise, 1 × 10^−8^.

To identify cell lines with a stringent ICRF193-imposed arrest suitable for a genome-wide siRNA screen we interrogated a panel of cell lines for their response to ICRF193 and used the DNA damaging agent Bleomycin as a positive control for a G2 arrest. As a readout of G2 arrest we monitored the mitotic index (MI) upon treatment with increasing drug concentrations of either ICRF193 or Bleomycin. Simultaneous addition of the microtubule antagonist Nocodazole was employed to trap arrest evading cells in mitosis enabling their quantification by immunofluorescence (IF) staining for the mitotic marker MPM2 (19). While the normal diploid cell line RPE1 and some cancer cell lines elicited a robust arrest in response to either drug, multiple cancer cell lines showed an impaired response to ICRF193 while retaining a response to Bleomycin (Figure 1A). Of note in arresting cells, Bleomycin but not ICRF193 caused a profound increase of the DNA damage marker γH2AX under these conditions (Supplementary Figure S1A). Combined, this suggests that distinct signalling pathways govern the DNA damage checkpoint and the Topo2a-dependent G2 arrest. Based on these cell profiles we selected RPE1 cells for a genome-wide siRNA screen. Using RPE1 cells we determined that there was no loss of viability in the 18 h arrest assay configured for the screen and further that the arrest observed with ICRF193 was not influenced by Nocodazole treatment. Reciprocally, we saw that ICRF193 does not significantly affect the nocodazole block, which was required to trap mitotic cells for G2 progression scoring.

Previously suggested effectors of the Topo2a-dependent arrest such as BRCA1, WRN, MDC1, ATM, ATR and Chk1 (5,6,13–16) were tested in RPE1 cells and none were found to have a non-redundant function in this arrest (data not shown and Supplementary Table S3). However, we noted that the combined knock-down of ATM and ATR elicited a profound arrest failure (Supplementary Figure S1B), consistent with their inhibition by caffeine (5). This combination knock-down was employed as a positive control for the screen, where a genome-wide siRNA library was screened for siRNAs that abrogated the ICRF193-induced G2 arrest (Supplementary Figure S1C). A stringent screening procedure was adopted (Supplementary Figure S1D) and candidate genes were selected on the basis of a significant arrest bypass, represented as the MI score (see Methods for hit selection criteria). Toxic siRNAs were identified by a decrease in the cell number and excluded from further analysis. Internal validation of the screen was provided by the fact that the previously identified arrest effector Topo2a was a strong hit (Figure 1B).

From the primary screen 317 potential hits were taken forward (Figure 1B, Supplementary Figure S1E and Supplementary Table S3) and we performed a secondary screen including further controls. 151 siRNAs (47.6%) repeated under the same assay conditions and six siRNAs (1.9%) were eliminated from subsequent analysis because they caused significant mitotic accumulation without ICRF193 treatment (Supplementary Figure S2A and Supplementary Table S4). The effects of the 317 candidates on the ICRF193-induced arrest were also validated in U2OS cells, a different cell line with a robust Topo2a-dependent arrest, (Supplementary Figure S2B and Supplementary Table S4). In U2OS cells 115 siRNAs reproduced the effect seen in RPE1 cells corresponding to 76.2% of the 151 repeated siRNAs. To investigate directly whether candidate hits specifically regulated only the Topo2a-dependent arrest we also implemented a counter-screen to interrogate the effect of the 317 primary hit siRNAs on the ICRF193- and the Bleomycin-induced arrests. Interestingly, 138 of the 151 repeated ICRF193 scoring siRNAs (92.7%) abrogated only the ICRF193- and not the Bleomycin-induced G2 arrest (Supplementary Figure S2C and Supplementary Table S4). These results indicate that the implementation of the DNA damage-induced G2 arrest and of the Topo2a-dependent G2 arrest are at least in part mechanistically distinct.

The 138 ICRF193 arrest selective candidates were subjected to siRNA deconvolution analysis, in which we individually tested the four oligonucleotide duplexes comprising the siRNA pools. The effect of an siRNA was considered likely to be on-target for a gene when two or more independent siRNAs against this gene abrogated the arrest. This was the case for 48 of the pools (35%) (Supplementary Figure S2D, Supplementary Table S5). To further investigate the notion that DNA damage and catalytic inhibition of Topo2a activate molecularly distinct regulatory processes, we repeated and extended the counter-screen including not only Bleomycin but also two additional treatments: the Topo2 poison Etoposide and γ-irradiation. Here, 100 genes consistently scored for ICRF193 and for none of the three DNA damage-inducing treatments (Figure 1C, Supplementary Figure S2E, Supplementary Table S6), while all treatments caused accumulation of cells in G2 (Supplementary Figure S2F). These results corroborate the conclusion that the ICRF193-induced arrest is regulated through mechanisms different from those involved in the DNA damage-induced arrest.

Protein-protein interactions between the 48 deconvoluted hits (Supplementary Figure S2D, Supplementary Table S5) were visualized using the STRING database (http://string-db.org/). To identify enriched networks and further potential interactors, hit candidates from the primary screen were sequentially added 10 at a time in a ranked *z*-score dependent manner. An additional 30 genes were included whilst maintaining statistical enrichment when compared to an equivalent number of randomly selected genes. The largest identified network contained Topo2a and the SMC5/6 complex, with 12 nodes (genes) and 24 edges (interactions) from the deconvoluted 48 hits. This was subsequently enriched to 18 nodes and 35 edges with the additional 30 proteins included (Figure 1D, Supplementary Figure S2G). The connectivity and significant enrichment of this network, alongside the predicted interaction with Topo2a, identified the SMC5/6 complex as a strong candidate involved in controlling the timing of the Topo2a-dependent G2 arrest.

### The SMC5/6 complex regulates the Topo2a-dependent G2 arrest, sister chromatid disjunction and recruits NSE2 to Topo2a

**Figure 2.**
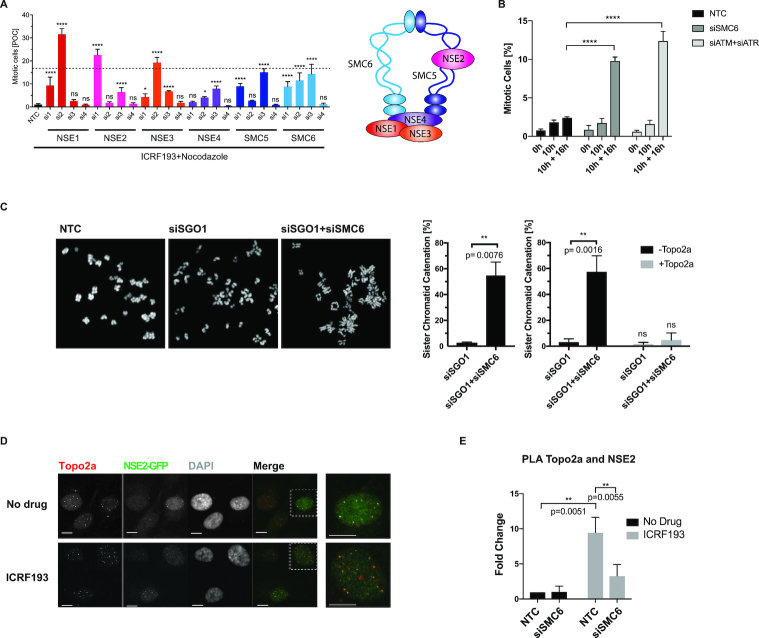
The SMC5/6 complex regulates the Topo2a-dependent G2 arrest, sister chromatid disjunction and recruits NSE2 to Topo2a. (**A**) Asynchronous RPE1 cells were transfected with the indicated siRNAs, treated with 3 μM ICRF193 and 1 μM Nocodazole for 18 h and the MI were quantified and expressed as percentage of control (POC), in line with the Deconvoluted screen as described in the materials and methods (left panel). Data are represented as mean ± S.D. and are compared to the average siATM+siATR positive control MI of 16.7% with an S.E.M of 0.61 from *n* = 48 triplicate repeats, indicated by the dotted line. Schematic representation of the SMC5/6 complex (right panel). (**B**) U2OS cells were transfected with non-targeting control (NTC), SMC6 or siATM+siATR siRNA, synchronized with a double thymidine block, released for 10 h and then treated with 3 μM ICRF193 and 1 μM Nocodazole for a further 16 h. The mitotic index was determined through flow cytometry and staining for PI and MPM2. Data were normalized to 16 h of 1 μM Nocodazole alone and represented as mean ± S.D. *n* = 3. (**C**) Confocal images of chromosome spreads of RPE1 cells transfected with the indicated siRNAs with and without incubation of recombinant Topo2a *in vitro*. Bar graphs show quantification of SCI. Data are represented as mean ± S.E.M. (**D**) IF staining of RPE1 cells for NSE2-GFP and Topo2a after treatment for 18 h with 3 μM ICRF193 as indicated. White dotted boxes highlight enlarged images displayed on the right. Scale bar = 10 μm. (**E**) MATLAB-aided quantification, as described in the IF image analysis section of the Materials and Methods, of PLA experiments using antibodies for Topo2a and NSE2-GFP. RPE1 cells were transfected with non-targeting control (NTC) or siSMC6 for 55 h and treated with 3 μM ICRF193 for a further 18 h. Data are represented as mean ± S.E.M.

In mammalian cells, the SMC5/6 complex consists of the SMC5/6 heterodimer and the non-SMC subunits NSE1–4 (Figure 2A). All subunits except for NSE4 scored in multiple screening rounds. NSE4, eliminated at the primary screen stage, was retested and upon deconvolution of its siRNA pool, two of the four oligos were seen to abrogate the ICRF193-induced arrest (Figure 2A). The deconvolution of the siRNA pool targeting SMC6 confirmed the ICRF193-induced override and the efficiency of the knock-down, through both real-time PCR and western blot (WB) (Supplementary Figure S3A). For further validation, we engineered a squamous lung cancer cell line H2170 with low SMC6 levels using a stably transfected shRNA, which equally impaired the ICRF193-induced arrest while retaining the DNA damage-induced G2 arrest (Supplementary Figure S3B). Interestingly, examination of publicly available patient data revealed a correlation between very low transcript levels of SMC5/6 and rapid disease progression in squamous lung cancer patients, indicating an association of complex depletion/loss with more aggressive tumours (Supplementary Figure S3C).

To confirm the specific G2 phase requirement of the SMC5/6 complex in response to compromised Topo2a activity, U2OS cells were synchronized in early S phase and the ICRF193-induced G2 arrest competency was analysed in the presence and absence of SMC6. ICRF193 and nocodazole were administered when the cells reached G2 phase (Supplementary Figure S3D) and we show that the G2 arrest is abrogated when either SMC6 or ATM and ATR levels are depleted (Figure 2B).

A recent publication suggested that loss of SMC6 causes mislocalization of Topo2a on mitotic chromosomes, with the presence of curly chromatids and distal enrichment (20). We observed this in some cases when performing an siRNA-mediated knockdown of SMC6, however we predominantly saw a more dramatic effect with a loss of Topo2a from mitotic chromosomes in RPE1 cells (Supplementary Figure S4A). This difference in observation compared to Gallego-Paez *et al.* (20) might be explained by the different fixation methods and antibodies used. Nevertheless, the conclusion that part of the function of the SMC5/6 complex is to assist the localization of Topo2a to chromosomes in mitosis is clear. Employing a premature chromosome condensation assay (21), we were able to extend this finding to G2-like chromosomes (Supplementary Figure S4B). Knockdown of SMC6 did not affect the expression levels of Topo2a (Supplementary Figure S4C), suggesting that SMC5/6 is not required for its stability. Using co-immunoprecipitation we detected an interaction between Topo2a and SMC6 (Supplementary Figure S4D), as recently shown by Verver *et al.* (22). To determine whether there was a direct interaction, the lysates were treated with benzonase prior to immunoprecipitation to remove chromatin and RNA, which might otherwise bridge the proteins (data not shown). We saw a small increase in this interaction with the addition of ICRF193, but this did not reach statistical significance (*P* = 0.1359). Using proximity ligation assays (PLA), this interaction was confirmed implying that the SMC5/6 complex and Topo2a interact closely (Supplementary Figure S4E), within 40 nm. To investigate the functional importance of this interaction we determined sister chromatid catenation by a previously established assay that monitors sister chromatid intertwines (SCIs) by removal of centromeric cohesion and viewing the chromosome formations (8). Briefly, knockdown of Sgo1 causes loss of sister chromatid cohesion, as Sgo1 is required to protect premature cleavage of centromeric cohesin in mitosis (23). After additional knockdown of SMC6, sister chromatids appeared to have incomplete arm disjunction where they were often seen held together at their arms or giving a ‘closed-arm’ appearance. Incubation with recombinant Topo2a reversed the observed tethering phenotype indicating that the tetherings indeed result from catenation (Figure 2C).

In order to understand how the SMC5/6 complex might promote Topo2a activity we then turned to the NSE2 subunit of the SMC5/6 complex. We hypothesized, that in addition to the above described SMC6-dependent recruitment of Topo2a, a possible signalling function could be elicited by the E3 SUMO ligase NSE2 in arrest implementation and/or sister chromatid resolution. To investigate a possible interaction between Topo2a and NSE2 we engineered RPE1 cells stably expressing NSE2-GFP as several antibodies tested were not able to detect endogenous NSE2. Using antibodies against Topo2a and GFP, colocalization of Topo2a and NSE2 was revealed in discrete foci (Figure 2D), which is in agreement with previous studies where NSE2 showed a punctate localization pattern (24). The high background observed in the NSE2-GFP signal precluded direct quantification and complementary PLA experiments were performed. Positive PLA foci further revealed that there was an interaction between Topo2a and NSE2 and quantification using MATLAB (as described in the Materials and Methods) showed these discrete foci increased in response to ICRF193, dependent upon the presence of SMC6. This suggested Topo2a as a possible ICRF193-dependent substrate of NSE2 (Figure 2E).

### Topo2a colocalizes with PML bodies and SUMO2/3 foci

**Figure 3.**
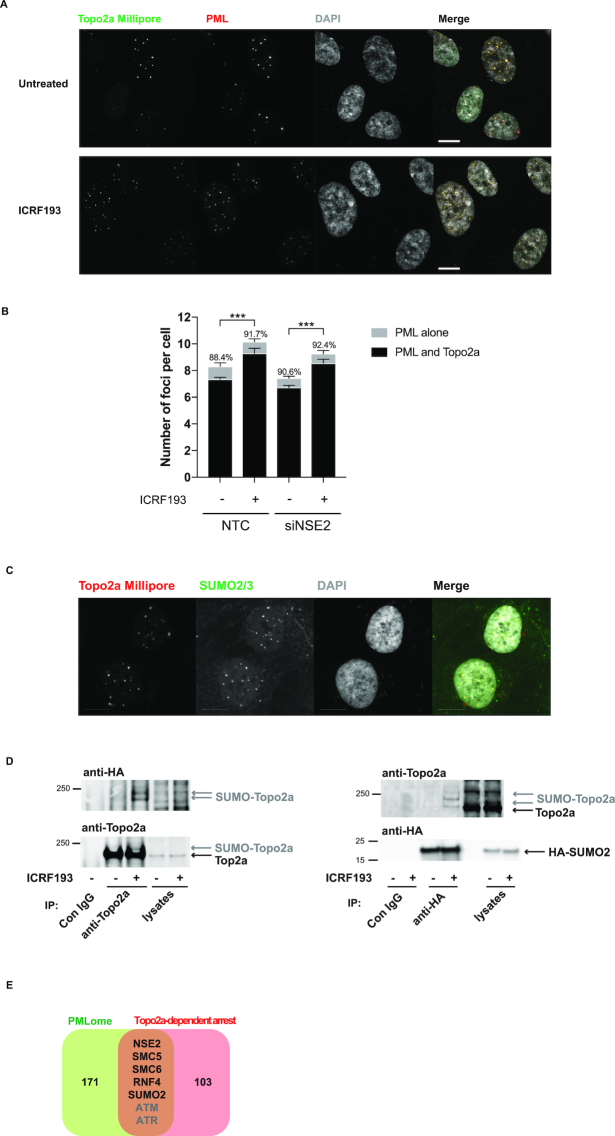
Topo2a colocalizes with PML bodies and SUMO2/3. (**A**) IF staining of RPE1 cells for Topo2a and PML after 18 h treatment with 3 μM ICRF193 as indicated. (**B**) MATLAB-aided quantification, as described in the IF image analysis section of the Materials and Methods, of colocalization between Topo2a (Millipore) and PML (abcam) in the presence and absence of NSE2. RPE1 cells were transfected with non-targeting control (NTC) or siNSE2 for 48 h and subsequently treated with 3 μM ICRF193 for 18 h where indicated. The percentages show the proportion of PML foci that were positive for Topo2a. Data represents mean ± S.E.M. for *n* = 3. (**C**) IF staining of RPE1 cells for SUMO2/3 and Topo2a. Scale bar = 10 μm. (**D**) RPE1 cells were transfected with HA-SUMO2, 24 h later treated with 3 μM ICRF193 for 18 h as indicated before lysates were subjected to either anti-Topo2a (left) or anti-HA (right) immunoprecipitation. Subsequent WB were performed for HA-SUMO and Topo2a. (**E**) Venn diagram showing overlap between the ICRF193-selective hits and a recently identified PMLome (31). ATM and ATR are redundant players in the Topo2a-dependent G2 arrest.

Topo2a foci have been described previously (14,25), but the nature of these nuclear bodies is not known. Given the colocalization of Topo2a with the SUMO E3 ligase NSE2, we assessed whether these foci were PML bodies, whose principal components are SUMO-modified and to which NSE2 had previously been shown to localize (18,26). IF analysis of RPE1 cells stained for Topo2a and PML, revealed specific colocalization of Topo2a foci and PML bodies (Figure 3A). MATLAB-aided quantification showed the number of these PML and colocalized foci increased significantly with ICRF193 treatment, but was not dependent upon the presence of NSE2 (Figure 3B). Topo2a foci were also found to colocalize with SUMO2/3 (Figure 3C) and as reported elsewhere (27), we observed ICRF193-induced SUMOylation of Topo2a (Figure 3D). We transfected RPE1 cells with HA-tagged SUMO2, immunoprecipitated either Topo2a or HA-SUMO2 and then probed with anti-HA and anti-Topo2a antibodies, revealing an enrichment of higher-molecular weight SUMO2-conjugated Topo2a in addition to unmodified Topo2a. SUMOylation of Topo2a has been detected in budding yeast, *Xenopus* egg extracts and human and murine cells during mitosis (27–30). To test whether the observed SUMOylation was cell cycle phase dependent or specifically associated with an ICRF193 induced arrest, cells were treated with Bleomycin to arrest them in G2. No increase in Topo2a SUMOylation was seen upon addition of Bleomycin, indicating that the observed ICRF193-triggered SUMOylation is not due to an arrest in G2 *per se* (Supplementary Figure S5A). As expected, the abundance of SUMO2-modified Topo2a was reduced when RPE1 cells were lysed in the absence of the alkylating agent and isopeptidase inhibitor iodoacetamide (IAA), which preserves SUMOylation through inhibiting SUMO-specific proteases (Supplementary Figure S5A).

Consistent with our findings, comparison of the ICRF193-selective hit list with a recently published PML-interactome (31) revealed several shared components, most of which are involved in SUMOylation, suggesting the Topo2a-dependent G2 arrest is regulated by SUMOylation at PML bodies (Figure 3E). This is further corroborated by the finding that the only known SUMO E1 enzyme, SAE1, was a strong hit in all screening rounds (Supplementary Tables S3-S6).

### Germline mutations demonstrate that NSE2 activity is required for the Topo2a-dependent G2 arrest

**Figure 4.**
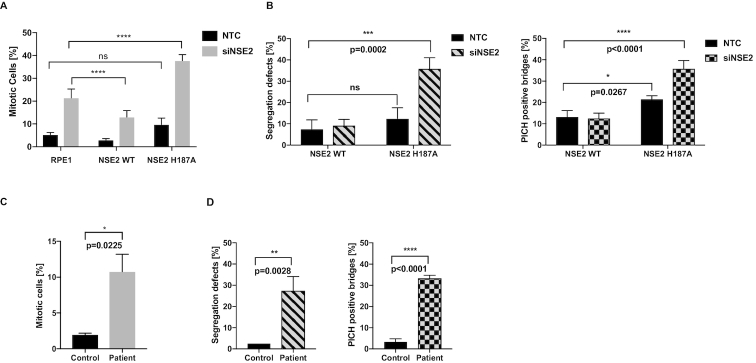
Germline mutations demonstrate that NSE2 activity is required for the Topo2a-dependent G2 arrest. (**A**) Quantification of MIs by automated IF analysis of parental RPE1 cells and RPE1 cells stably expressing NSE2wt-GFP (NSE2 WT) or the ligase dead mutant NSE2H187A-GFP (NSE2 H187A). Cells were transfected with the indicated siRNAs and treated with 3 μM ICRF193 and 1 μM Nocodazole for 18 h, fixed and stained for MPM2 and DAPI. The siNSE2 transfection caused a significant increase (*P* < 0.0001) in mitotic cells compared to NTC (non-targeting control) for each cell line. Data are normalized to 1 μM Nocodazole alone and are represented as mean ± S.D. of a representative experiment of *n* = 3 with six technical replicates. (**B**) RPE1 cells stably expressing siNSE2-resistant NSE2wt-GFP or the ligase dead mutant NSE2H187A-GFP transfected with the indicated siRNAs were 48 h later fixed and stained for PICH and DAPI. Quantification of segregation defects as defined by chromatin bridges or lagging chromosomes and PICH-positive ultrafine bridges. Data are represented as mean ± S.D. A two-tailed *t*-test was used to analyse statistical significance. *n* = 3 with each 40 cells. (**C**) Quantification of MIs by FACS analysis of control and patient fibroblasts treated with 3 μM ICRF193 and 1 μM Nocodazole for 24 h, fixed and stained for MPM2 and PI. Data are normalized to 1 μM Nocodazole alone and are represented as mean ± S.D.. A two-tailed *t*-test was used to analyse statistical significance. *n* = 3. This data is part of Supplementary Figure S5D. (**D**) Control and patient fibroblasts were fixed and stained for PICH and DAPI. Quantification of segregation defects as in (B).

To address whether the ligase activity of NSE2 regulated the Topo2a-dependent arrest and/or sister chromatid resolution, we initially used stable RPE1 cell lines expressing siRNA-resistant NSE2wt-GFP or the ligase dead mutant NSE2-H187A-GFP (32). The expression of NSE2wt-GFP partially recovered the G2 arrest, however the NSE2 inactive mutant exhibited a dominant negative behaviour where it further increased the percentage of mitotic cells (Figure 4A) and also interfered with the ability to correct chromosome bridges. This is shown by an increase in DAPI-positive chromatin bridges, an increase in lagging chromosomes and an increase in PICH-positive ultrafine bridges (Figure 4B, Supplementary Figure S5B), the last of these is a property suggested to reflect catenation between sister centromeres (33,34).

To validate independently whether NSE2 dysfunction influences these properties, we sought to test cells from patients with rare germline mutations in NSE2 resulting in severely depressed levels of full-length NSE2 (17). A remarkably consistent effect was seen with a compromised Topo2a-dependent G2 arrest response (Figure 4C) and a profound increase in segregation errors and PICH-positive ultrafine bridges (Figure 4D, Supplementary Figure S5C). Expression of wildtype NSE2 in these patient cells, but not the ligase-dead NSE2 mutant, restored the functional arrest corroborating its dependence on the ligase activity of NSE2 (Supplementary Figure S5D).

### NSE2-mediated SUMOylation of Topo2a is essential for Topo2a-dependent sister chromatid disjunction

**Figure 5.**
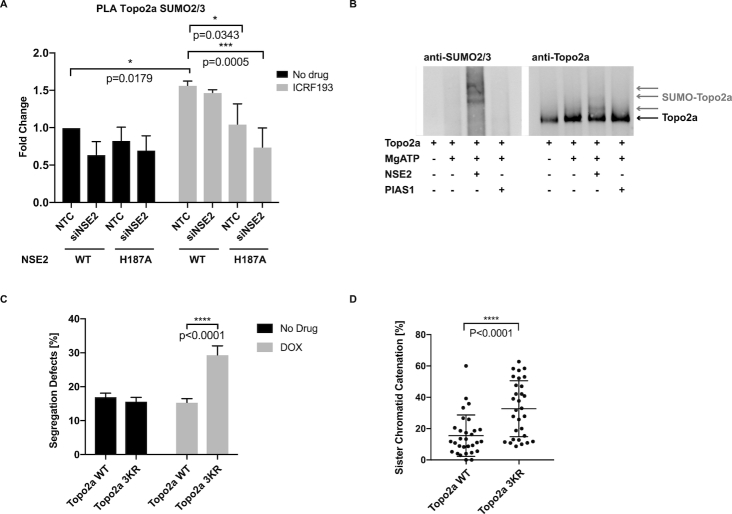
NSE2-mediated SUMOylation of Topo2a is essential for Topo2a-dependent sister chromatid disjunction. (**A**) MATLAB-based quantification of PLA experiments using antibodies against Topo2a and SUMO2/3. RPE1 cells stably expressing NSE2wt-GFP or the ligase dead mutant NSE2H187A-GFP were transfected with the indicated siRNAs and treated with 3 μM ICRF193 for 18 h. Data are represented as mean ± S.E.M. (**B**) WB of *in vitro* SUMOylation assays using recombinant human Topo2a, SAE1, UBC9, SUMO2 and SUMO3 incubated for 30 min at 37°C with NSE2-GST or PIAS1-GST as indicated. See also Supplementary Figure S6C and D. (**C**) U2OS FlpIn cells stably expressing inducible Topo2awt-GFP or Topo2a3KR-GFP induced for 48 h with Doxycycline as indicated, fixed and stained with DAPI. Quantification of segregation defects as defined by chromatin bridges or lagging chromosomes. Data are represented as mean ± S.D. *n* = 3 each with 50 cells. (**D**) U2OS FlpIn cells as in (C) induced for 24 h with Doxycycline and transfected with siSGO1. The graph shows quantification of SCI in at least 28 cells. Data are represented as mean ± S.D. of a representative experiment. *n* = 3.

Given the ICRF193-triggered increase of colocalized Topo2a and NSE2 foci (Figure 2D, E), we determined whether the ICRF193-induced SUMOylation of Topo2a (Figure 3D) was dependent on NSE2. We analysed Topo2a SUMOylation by PLA using antibodies against endogenous Topo2a and SUMO2/3 in stable cell lines expressing NSE2wt or NSE2-H187A. Quantification of the PLA foci confirmed not only an ICRF193-dependent increase of Topo2a SUMOylation, but also established its dependence on NSE2 (Figure 5A). Knockdown of SMC6 equally abrogated the ICRF193-triggered SUMOylation of Topo2a (Supplementary Figure S6A). Co-immunoprecipitation experiments showed that NSE2-H187A still interacts with SMC6 and hence the decreased SUMOylation is not due to aberrant complex formation (Supplementary Figure S6B). To test whether Topo2a is a direct substrate for SUMO modification by NSE2, we investigated SUMOylation *in vitro* using purified human Topo2a and recombinant NSE2 in the presence of SAE1 and UBC9. Topo2a SUMOylation was analysed by immunoblotting for Topo2a and SUMO2/3. As shown by the increase in SUMO2/3 conjugates of high molecular mass (>170kDa) (Figure 5B), Topo2a was SUMOylated by NSE2, whereas no such modification was observed if NSE2 was replaced by the SUMO E3 ligase PIAS1. We found that Topo2a was also SUMOylated *in vitro* by PIAS4 (Supplementary Figure S6C), which had been suggested previously to mediate SUMOylation of Topo2a specifically in mitosis (27,29). NSE2 shows specificity in these reconstitution experiments as it did not promote the SUMOylation of RanGP1 (Supplementary Figure S6D).

To address the functional importance of this NSE2-mediated SUMOylation we sought to map the modification site(s) on Topo2a. In order to do so, we took advantage of the observation that two antibodies raised against the very C-terminus of Topo2a (antibody #1 and #2) did not recognize the slow-migrating bands of SUMOylated Topo2a seen in response to ICRF193. These bands were only recognized by antibody #3 that was raised against full-length Topo2a (Supplementary Figures S7A, B). We therefore speculated, that SUMOylation might compromise the C-terminal epitope(s) recognized by these antibodies. A peptide consisting of the last 18 amino acids of Topo2a recognized by antibody #1 and #2 but not by antibody #3 contained three lysines presenting potential SUMOylation sites (Supplementary Figure S7B). Indeed, branched peptides, in which the five C-terminal amino acids of activated SUMO2/3 were attached to each of the potential acceptor lysines to mimic SUMOylation at these sites, interfered to varying extents with the recognition by antibodies #1 and #2 (Supplementary Figure S7C). We therefore considered the possibility that all three lysines may become SUMOylated and engineered U2OS FlpIn cells stably expressing either Topo2awt-GFP or mutant Topo2a^K1516,1517,1520R^ denoted Topo2a3KR-GFP. As anticipated this mutant was not recognized by a C-terminus directed antibody but was efficiently detected by the Topo2a-full-length or GFP recognizing antibody (Supplementary Figure S7D). Topo2a3KR mimicked expression of ligase-dead NSE2 causing an increase in chromosome bridges (Figure 5C), but interestingly this was not accompanied by an increase in PICH-positive ultrafine bridges (Supplementary Figure S7E). To determine whether SUMOylation of Topo2a at the C-terminus is important for Topo2a catalytic activity, we assessed sister chromatid catenation using the chromosome spread assay. As shown in Figure 5D, expression of Topo2a3KR caused a significant increase in sister chromatid catenation indicating compromised Topo2a activity of this mutant.

### K1520 is a novel NSE2 SUMOylation site on Topo2a

**Figure 6.**
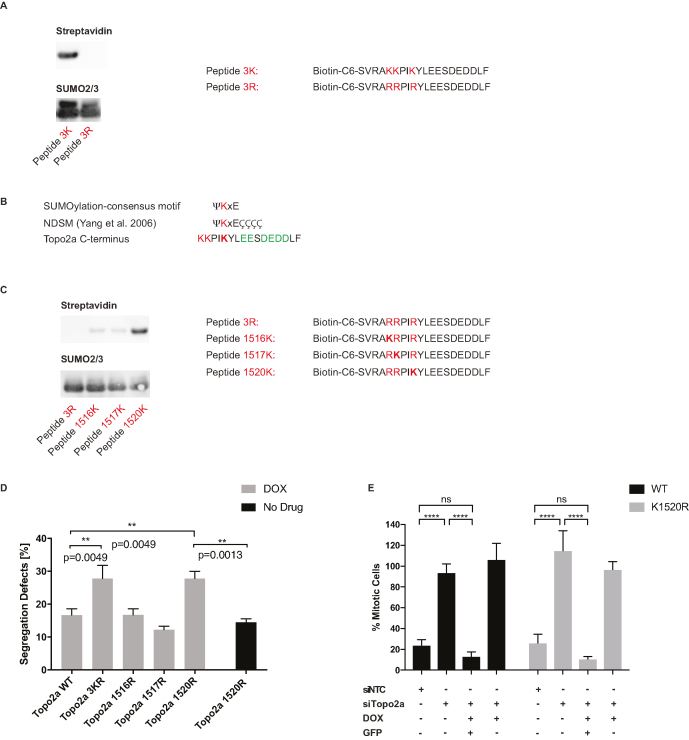
K1520 is a novel NSE2 SUMOylation site on Topo2a. (**A**) WB of *in vitro* SUMOylation assays using purified peptides as indicated and SAE1, UBC9, SUMO2 and SUMO3 incubated for 45 min at 37°C with NSE2-GST. The two bands on the SUMO2/3 blot correspond to SUMO2 and SUMO3, which have different molecular weights. Only SUMOylated peptides are large enough to be retained on the SDS-PAGE gel, for transfer and detection by streptavidin-HRP. (**B**) Comparison of the minimal and the extended SUMO-consensus (NDSM) site with the C-terminus of Topo2a, where ψ represents an amino acid with an alipathic side chain and Ç represents a negatively charged amino acid. (**C**) Peptide SUMOylation *in vitro* as in (A). (**D**) U2OS FlpIn cells stably expressing Doxycycline-inducible GFP-tagged Topo2a, Topo2a^K1516R^, Topo2a^K1517R^, Topo2a^K1520R^ or Topo2a3KR induced for 48 h with Doxycycline as indicated, fixed and stained with DAPI. Quantification of segregation defects as defined by chromatin bridges and lagging chromosomes. Data are represented as mean ± S.D. *n* = 3 each with 30 cells. (**E**) U2OS FlpIn cells stably expressing doxycycline-inducible GFP-tagged Topo2a WT or Topo2a^K1520R^ were induced for 48 h with Doxycycline and transfected with siRNAs as indicated. Cells were synchronized by a double thymidine block and 5 h after release were treated for 24 h with 3 μM ICRF193 and 1 μM Nocodazole. After fixation cells were stained for GFP, MPM2 and PI. Data are normalized to 1 μM Nocodazole alone and doxycycline induced cells were gated for a GFP positive and negative population, where there were over 1000 GFP positive cells, per condition in each experiment. Data are represented as mean ± S.D. *n* = 3.

To assess where NSE2 SUMOylates Topo2a, a biotinylated peptide covering the last 20 amino acids at the C-terminus of Topo2a (Topo2a^1512–1531^, denoted peptide 3K) was synthesized alongside a modified peptide, in which all the lysines were replaced by arginine (peptide 3R). A SUMOylation assay in the presence of recombinant NSE2 was performed and after separation by SDS-PAGE the sumoylated, biotinylated peptides were identified by probing with Streptavidin–HRP (note the free peptides were run off the gel). As shown in Figure 6A, the 3K but not the 3R peptide was efficiently SUMOylated by NSE2.

Interestingly, none of the lysines in this C-terminal sequence conform to the minimal SUMOylation consensus motif ΨKxE (Figure 6B). However, an extended SUMOylation motif, termed negatively charged amino acid-dependent SUMOylation motif (NDSM) was recently reported, suggesting an overrepresentation of acidic amino acids downstream of a putative SUMOylation site with a concentration at positions 3–6 (35). Comparing the NDSM with the C-terminus of Topo2a, we hypothesized that K1520 might be the preferred SUMOylation site as there are multiple glutamates and aspartates at position 3, 4 and 6–9 downstream of K1520 (Figure 6B). Indeed, when using peptides in which pairs of lysines were replaced by arginine a clear preference for SUMOylation of K1520 was seen (Figure 6C). Subsequent generation of SUMOylation-deficient single site mutants (Topo2a^K1516R^, Topo2a^K1517R^, Topo2a^K1520R^) confirmed that only the K1520R mutation phenocopies the segregation defect seen in the Topo2a3R mutant (Figure 6D). These findings identify a novel, non-canonical SUMOylation site in Topo2a that is modified in response to compromised Topo2a activity.

Given that NSE2 and its E3 ligase activity are essential to implement the Topo2a-dependent G2 arrest (Figures 2A and 4A), we assessed whether the novel NSE2-mediated SUMOylation site K1520 was also required for this arrest mechanism. In polyclonal populations of synchronized G2 arrest competent U2OS cells, inducible expression of either Topo2awt-GFP or Topo2a^K1520R^ in combination with siRNA mediated knockdown of endogenous Topo2a fully recovered the ICRF193 induced G2 arrest (Figure 6E). Sorted cells induced but not expressing Topo2a-GFP within these polyclonal populations were unable to recover the G2 arrest providing an internal control to the observed rescue. This indicates that there are both arrest and resolution signals emanating from NSE2 E3 SUMO ligase activity as part of the SMC5/6 complex, with only the latter signal being relayed by K1520 SUMOylation of Topo2a.

## DISCUSSION

Topo2a-mediated resolution of sister chromatid intertwinings is crucial for correct chromosome segregation and thus genetic integrity. Our analysis of the Topo2a-dependent arrest and reports by others (8,10,12,36) show that this arrest is frequently attenuated or inactivated in cancer cells, suggesting caution should be exercised when drawing conclusions about the associated mechanisms operating in transformed cells. There is however a need for a deeper understanding of this arrest mechanism since this might provide opportunities for therapeutic intervention, through targeting the emergent failsafe pathways (8). Here, we set out to characterize the non-redundant genetic requirements for the Topo2a-dependent G2 arrest in non-transformed cells using an unbiased siRNA screen.

Previous studies implicated BRCA1, WRN, MDC1, ATM, ATR, Chk1 and Topo2a as players in this ICRF193-induced arrest (5,6,13–16). The present study confirms the involvement of Topo2a as well as of ATM+ATR (identified as redundant players in pre-screening studies). However, in both the primary screen as well as when tested independently, we failed to see an impact on G2 arrest in these normal epithelial cells upon loss of BRCA1, Chk1, WRN or MDC1 using siRNA-mediated knockdown. Of note, almost all of the studies describing these putative arrest players used transformed cells, more precisely DT40 (13) and HeLa cells (6,14), which both have an attenuated response to ICRF193 (16). This may indicate the action of redundant functions, wherein components emerge as essential in transformed somatic variants with compromised redundant pathways. However, we do not exclude the possibility that insufficient knockdown or differences in experimental setup as highlighted by Bower *et al.* might explain these discrepancies (16).

The family of SMC proteins, which comprises SMC1/3 (Cohesin), SMC2/4 (Condensin) and SMC5/6, play key roles in the maintenance of genome integrity through their function in DNA replication, segregation and repair. Whereas the functions of Cohesin and Condensin are well established, it has proven difficult to ascertain how the SMC5/6 complex safeguards chromosome stability (37–39). Several groups have shown that the complex has a role in DNA repair through homologous recombination (HR) and that it resolves or prevents aberrant recombination intermediates. However, the consensus is that the SMC5/6 complex is likely to have additional functions due to its essential requirement in yeast, where other recombination proteins are dispensable for survival (37,39–41). Indicative of distinctive, hitherto undefined actions for this complex beyond DNA repair and stalled replication fork resolution, cell cycle regulated alleles restricting expression of SMC5/6 components revealed that the complex is essential in G2/M but not in S-phase (42).

It has been speculated that the SMC5/6 complex concentrates around different types of joint molecules, such as SCI, stalled forks and DNA repair intermediates, and recruits complexes involved in their resolution (43). Murine and human cells depleted of SMC5 or SMC6 accumulate chromatid-linking anaphase bridges (20,44). Also, Verver *et al.* observed that NSE2 null cells showed reduced survival upon treatment with Etoposide and that SMC6 interacted with Topo2. Combining these results, they hypothesized that NSE2 helps resolve topological stress (22). An elegant study in budding yeast concluded that SMC5/6 possibly associates with SCIs and facilitates their resolution (45,46). By contrast, Kanno *et al.* suggested that SMC5/6 sequesters these catenanes behind the fork thereby facilitating fork rotation and resolution of superhelical tension (47). This finding is however inconsistent with the hallmark of segregation problems for the knockdown phenotype, which indicates a function for SMC5/6 in the resolution of SCI. Moreover, SMC5/6 and NSE2 hypomorphs are synthetically lethal with mutants of Topo2 (48), which is not essential for the resolution of supercoils but only for the resolution of SCI (45).

Here, we uncover an essential role for the SMC5/6 complex in the Topo2a-dependent G2 arrest and in sister chromatid resolution. On ICRF193-induced G2 arrest we show increased interaction of Topo2a and the SMC5/6 subunit NSE2 at PML bodies, which are known nuclear SUMOylation hotspots that have previously been implicated in the response to cellular stress (31,49). We find that although NSE2 is not required for Topo2a localization at PML bodies, its E3 SUMO ligase activity is crucial to yield a Topo2a and SUMO2/3 proximity signal. This observation, alongside the finding that NSE2 supports Topo2a SUMOylation *in vitro* leads us to conclude that NSE2 SUMOylates Topo2a at PML bodies in an ICRF193-dependent manner. We map the NSE2-dependent SUMOylation to a previously unrecognized, non-canonical site, K1520, on Topo2a.

We, and others, show Topo2a localization on chromosomes is dependent on the SMC5/6 complex (20), yet we demonstrate SMC5/6 loss does not cause a prolonged ICRF193-induced G2 arrest accompanying decreased chromatin-associated Topo2a activity. Instead, the SMC5/6 complex is essential for G2 arrest implementation and cells expressing ligase-dead NSE2 or Topo2a lacking the C-terminal SUMO acceptor site K1520 have impaired chromosome segregation reminiscent of catalytic Topo2a inhibition (50). These observations are consistent with the requirement for Topo2a itself in this arrest and with several recent studies that show the C-terminus of Topo2a is particularly important for the regulation of the Topo2a-dependent arrest in yeast and human cells (6,7,51). Furthermore, it is also in agreement with its previously proposed requirement for faithful chromosome segregation in drosophila (52).

The SUMO pathway has emerged as an important determinant of genome stability but the underlying molecular mechanisms, acceptor residues and involved E3 ligases have proved difficult to define (53,54). SUMOylation of Topo2 was detected in mitotic extracts from yeast, *xenopus* egg extracts (XEE) and in murine embryo fibroblasts (MEFs). The combined evidence strongly suggests Topo2 SUMOylation to be important for chromosome segregation (28,30,55,56). In XEE the E3 ligase was identified as PIAS4 and acceptor residues were reported as K660, K1235, K1276 and K1298, however mutation of these did not completely block SUMOylation of Topo2 so additional sites must exist. Furthermore, PIAS4 was not shown to SUMOylate Topo2a directly in human cells (57) and PIAS4-/- mice have been found to be devoid of any overt phenotypes, whereas mice with low amounts of the E3 ligase RanBP2 developed severe aneuploidy (30). In line with this, RanBP2 was identified as critical for the SUMOylation of Topo2a at unidentified acceptor residues, but only in mitotic MEFs (57). Therefore, this questions the identity of the ligase involved in interphase. Interestingly, RanBP2 hypomorphic mice are prone to spontaneous tumours, as observed for mice with reduced expression of NSE2 (24). NSE2 heterozygous MEFs also presented signs of improper chromosome segregation such as micronuclei and polynucleated cells, but did not have major deficiencies in DNA repair or replication.

A recent report that characterized patients presenting reduced levels of NSE2 due to compound heterozygous frameshift mutations demonstrated that expression of NSE2 wt, but not an NSE2 ligase-dead mutant, restored nuclear abnormalities such as micronuclei and nucleoplasmic bridges (17). Here, we show that fibroblasts from these patients also exhibit a defective Topo2a-dependent G2 arrest and a dramatic increase in DAPI and PICH-positive anaphase bridges indicating reduced Topo2a functionality. In a previous study, knock-in mice with mutations that reduce/inactivate the SUMO ligase activity of NSE2 were shown to be physiologically normal (24). However, residual ligase activity was detected for the ‘ligase dead’ mutant protein (24), which may have been sufficient to sustain Topo2 function. Furthermore, unlike many ubiquitin E2 enzymes, UBC9 interacts with the SUMO modification consensus sites in target proteins directly, such that SUMO conjugation can occur without E3 enzymes in the presence of high concentrations of UBC9 (29). The elevation of local UBC9 concentrations at PML bodies together with partially active NSE2 might be sufficient to maintain Topo2 SUMOylation levels. It is also possible that in mice other PIAS family members can compensate for the absence of NSE2 to a greater extent than seems to be the case in humans. In yeast, the mild phenotype of NSE2 SUMOylation-deficient strains is greatly exacerbated upon deletion of additional SUMO ligases (24).

Despite the requirement of Topo2a, NSE2 and its E3 ligase activity for the implementation of the G2 arrest mechanism, we find the NSE2-mediated SUMOylation of Topo2a at K1520 is not essential to prevent mitotic progression but is important for sister chromatid resolution. We can conclude that there must be additional SUMOylation targets of NSE2 in response to compromised Topo2a activity that mediate the G2 arrest and that the SUMOylation of K1520 is required as part of a distinct resolution pathway.

Using a well validated screen, the present report has identified a wealth of non-redundant players in the operation of the Topo2a-dependent G2 arrest, among them the multifunctional SMC5/6 complex that includes the E3 ligase NSE2. We have demonstrated that NSE2 is a critical regulator in response to compromised Topo2a activity and that its ligase activity is required for a functional G2 arrest. Furthermore, we identified Topo2a as a novel target of NSE2 and reveal a non-canonical SUMOylation site required for efficient sister chromatid resolution. Although further mechanistic details are yet to be identified from the fruits of this genome-wide screen, the results here provide a significant advance in our understanding of the biological functions of the SMC5/6 complex. Furthermore, it has provided opportunities to determine how the SMC5/6 associated functions and those exerted by other hits interact to deliver an arrest, resolution and exit from this enigmatic, genome protecting, control point.

## Supplementary Material

Supplementary Data
