# Supplementary material for: A genome-wide RNAi screen identifies the SMC5/6 complex as a non-redundant regulator of a Topo2a-dependent G2 arrest
Source: Nucleic Acids Res. 2018 Dec 24;47(6):2906–21. doi: 10.1093/nar/gky1295 (PMC6451093; doi:10.1093/nar/gky1295)

Supplemental Figure Legends

Figure S1: A genome-wide RNAi screen for effectors of a Topo2a-dependent arrest

A) IF of RPE1 cells stained for γH2AX and DAPI (upper panel) and WB of RPE1 cell lysates stained for γH2AX and Tubulin (lower panel) after treatment of cells with 10µM Bleomycin or 3µM ICRF193 for 18h. B) Asynchronous RPE1 cells were transfected with non-targeting control (NTC) or siATM+siATR for 55hours and were subsequently treated with 3µM ICRF193 and 1µM Nocodazole or 3µM ICRF193, 1µM Nocodazole and 2mM Caffeine for 18hours. Subsequently cells were fixed and stained for MPM2 and DAPI. Data are represented as Mean ± S.D. when normalised to Nocodazole alone. A representative experiment with 6 technical replicates is shown. C) Screen protocol and assay principle to identify G2 arrest-defective cells. Asynchronous RPE1 cells were transfected in triplicate, 55hours later cells were treated for a further 18h with 3µM ICRF193 and 1µM Nocodazole, fixed and stained for the mitotic marker MPM2 and for DNA using DAPI. Shown are representative images acquired on the ArrayScan of cells transfected with non-targeting control (NTC) or with siRNAs against ATM and ATR exhibiting an active or defective arrest, respectively. The mitotic index (MI) was determined by automated IF analysis. Throughout the paper, each data point represents the median of three replicates of an individual siRNA pool. The same protocol was used for all screening rounds and assays. D) Overview of screening strategy and hit selection. E) Scatterplot of the log normalised MIs as measured on a laser scanning cytometer (Acumen) and an automated microscope (ArrayScan). Data acquired using the ArrayScan is also depicted in an alternative manner in Fig 1B. NT: non-targeting, RF: Risk-free.

Figure S2: The Topo2a-dependent G2 arrest is mechanistically distinct from the DNA damage checkpoint

A)-C) Scatterplots comparing the MI for the secondary hit library of RPE1 cells treated with 3μM ICRF193 + 1μM Nocodazole with those of untreated RPE1 cells (A), with those of 3μM ICRF193 + 1μM Nocodazole-treated U2OS cells (B) and with those of RPE1 cells treated with 10μM Bleomycin + 1μM Nocodazole (C) within the same counter-screen. Drug treatments were for 18h. D) Results of deconvolution of siRNA pools from the secondary screen in RPE1 cells. The SMC5/6 complex components are highlighted in red. E) MIs obtained in the extended counter-screen using the same library as in the second screen. Scatterplots compare the MIs of 3μM ICRF193 + 1μM Nocodazole treated RPE1 cells with those of no drug, 10μM Bleomycin + 1μM Nocodazole or 1μM Etoposide + 1μM Nocodazole treated cells and with those of RPE1 cells subjected to 10GY γ-irradiation and treated with 1μM Nocodazole. Drug treatments were for 18h. F) FACS analysis showing that treatment of RPE1 cells with 3μM ICRF193, 10μM Bleomycin, 1μM Etoposide or 10Gy γ-irradiation causes an accumulation of cells in G2. Drug treatments were for 18h. G) Summary results of nodes (genes) and edges (interactions) for each predicted network identified from the STRING database for the 48 deconvoluted hits and 30 additional primary screen hits, which were sequentially added in groups of ten before reaching the enrichment score threshold for background noise, $1x10^{-8}$.

Figure S3: Validation that the SMC5/6 complex is required for the Topo2a-dependent G2 arrest

A) RPE1 cells transfected with four different siRNAs targeting SMC6 or non-targeting control (NTC) siRNA. Bar graphs show the quantification of protein levels (upper left panel), mRNA levels (upper right panel), as determined by Western blot and RT-PCR, respectively, and mitotic index when treated with 3µM ICRF193 and 1µM Nocodazole for 18h (lower panel). Data are represented as Mean ± S.D.. B) WB of H2170 squamous lung cancer cell lysates stably expressing shRNAs targeting Luciferase (Luc) or SMC6 (upper left panel). Asynchronous H2170 cells were drug treated for 24h as indicated (lower left panel) and the mitotic indices were quantified through automated IF analysis and normalised to nocodazole only treatment, showing that chronic loss of SMC6 disables only the Topo2a-dependent arrest but not the DNA-damage-induced checkpoint. Data are represented as Mean ± S.D.. DNA profiles of H2170 cells treated with 1µM ICRF193 for 48h as indicated (upper right panel). Quantification of the percentage of cells arrested in G2 and of cells with a DNA content of >4N confirming that cells with low levels of SMC6 exit the Topo2a-dependent arrest with aberrant DNA contents. C) Kaplan-Meier survival curves of LUSC patient groups according to upper and lower pentile mRNA levels of SMC6, and SMC5 and/or SMC6. Statistical significance was tested using a log-rank test (upper panel). Boxplot of mRNA levels of SMC5 and SMC6 in LUSC patient groups according to pentile mRNA levels of SMC6, and SMC5 and/or SMC6. Data are represented as Mean ± S.D.. Statistical significance was tested using a t-test (lower panel). D) DNA profiles of U2OS cells transfected with non-targeting control (NTC) or siATM and siATR at the indicated timepoints after release from a double thymidine block. The respective percentage of mitotic cells is shown in the top right hand corner of each plot.

Figure S4: SMC6 interacts with Topo2a

A), B) Confocal images of mitotic (A) and G2-like (B) chromosome spreads from RPE1 cells transfected with non-targeting control siRNA (NTC) or targeting SMC6; cells are stained for Topo2a and DAPI. Cells were treated with 1µM Nocodazole (A) for 12h or with 50µM Calyculin A (B) for 30 min. Filled arrowheads indicate curly sister chromatids and open arrowheads distal localisation of Topo2a, higher magnification of examples are shown in the white dotted boxes. Scale bar=10µm. C) WB of RPE1 cell lysates transfected with the indicated siRNAs. D) WB showing co-immunoprecipitation of Topo2a and SMC6 in RPE1 cell lysates that had been treated with 0.02U/ml Benzonase for 1h, after treatment for 18h with 3µM ICRF193 as indicated. E) IF of RPE1 cells stained for Topo2a and SMC6 and subjected to PLA. Scale bar=20µm.

Fig S5: NSE2 SUMO ligase activity is required for chromosome segregation and arrest implementation

A) WB of co-immunoprecipitated HA-SUMO2 and Topo2a confirming ICRF193-induced SUMOylation of Topo2a. RPE1 cells were transiently transfected with HA-SUMO2, 24 hours later treated with 3µM ICRF193 or 10µM Bleomycin for 18h as indicated before lysates were subjected to anti-HA precipitation. The lysate buffer contained Iodoacetamide (IAA) to suppress desumoylation unless otherwise indicated. B&C) Confocal images of RPE1 cells (B) or healthy control and patient fibroblasts (C) used in Fig 4B and D stained for DAPI or PICH, showing normal mitoses and the types of segregation errors observed. Scale bar=5µm. D) Healthy control fibroblasts or patient fibroblasts with or without Doxycycline-induced expression of NSE2 wt or ligase dead NSE2 (H187A) were treated as indicated with Doxycycline for

24h and subsequently with 3µM ICRF193 and 1µM Nocodazole for 24h, fixed and stained for MPM2 and PI. MIs were quantified by FACS analysis and normalised to 1µM Nocodazole alone. Data are represented as Mean ± S.D. N=3.

Fig S6: Topo2a is specifically SUMOylated by NSE2

A) MATLAB-aided quantification of PLA experiments (see Material and Methods) using antibodies against endogenous Topo2a and SUMO2/3 showing an ICRF193-induced and SMC6-dependent SUMOylation of Topo2a in RPE1 cells treated with ICRF193 for 18h. Data are represented as Mean ± S.E.M.. B) WB showing co-immunoprecipitation of NSE2-GFP and SMC6 in RPE1 cells stably expressing NSE2wt-GFP or the ligase dead mutant NSE2H187A-GFP. The anti-NSE2 lysate blot shows a non-specific band around the size of NSE2-GFP (marked *), which is not detected after GFP-TRAP immunoprecipitation. C&D) WB of *in vitro* SUMOylation assays using recombinant human Topo2a (C) or RanGP1 (D) and SAE1, UBC9, SUMO2 and SUMO3 incubated for 30min at 37°C with NSE2-GST or PIAS1-GST or PIAS4-GST as indicated.

Fig S7: ICRF193 induces SUMOylation of Topo2a at the C-terminus

A&B) WB of RPE1 lysates after treatment of cells with ICRF193 for 18h incubated with anti-Topo2a antibodies #1 (Topogen), #2 (Abcam) or #3 (Millipore) in the absence of any peptides (A) or after incubation of antibodies with the indicated peptides at 1.5µg/ml for 1h at room temperature (B). C) WB of RPE1 lysates incubated with anti-Topo2a antibodies #1 (Topogen) or #2 (Abcam) after incubation of antibodies with the indicated peptides at 1.5µg/ml for 1h at room temperature. D) WB showing endogenous Topo2a and Doxycycline (DOX)-induced expression of GFP-tagged Topo2a-wt and Topo2a-3KR in U2OS-FlpIn cells. Doxycycline induction was for 48h.

E) U2OS FlpIn cells stably expressing inducible Topo2awt-GFP or Topo2a3KR-GFP induced for 48h with Doxycycline as indicated, fixed and stained with DAPI and PICH. Quantification of PICH-positive bridges.

Supplemental Tables:

Table S1: Reagents Table.

Table S2: Cell line information.

Table S3: Results of the Primary Screen, related to Fig 1. See Materials and Methods for hit criteria. Hits are highlighted in yellow.

Table S4: Results of the Secondary Screen, related to Fig 1. See Materials and Methods for hit criteria. Hits are highlighted in yellow.

Table S5: Results of the Deconvolution Screen, related to Fig 1. See Materials and Methods for hit criteria. Hits are highlighted in yellow.

Table S6: Results of the Extended-counter Screen, related to Fig 1. See Materials and Methods for hit criteria.

Fig S1: A genome-wide RNAi screen for effectors of a Topo2a dependent arrest

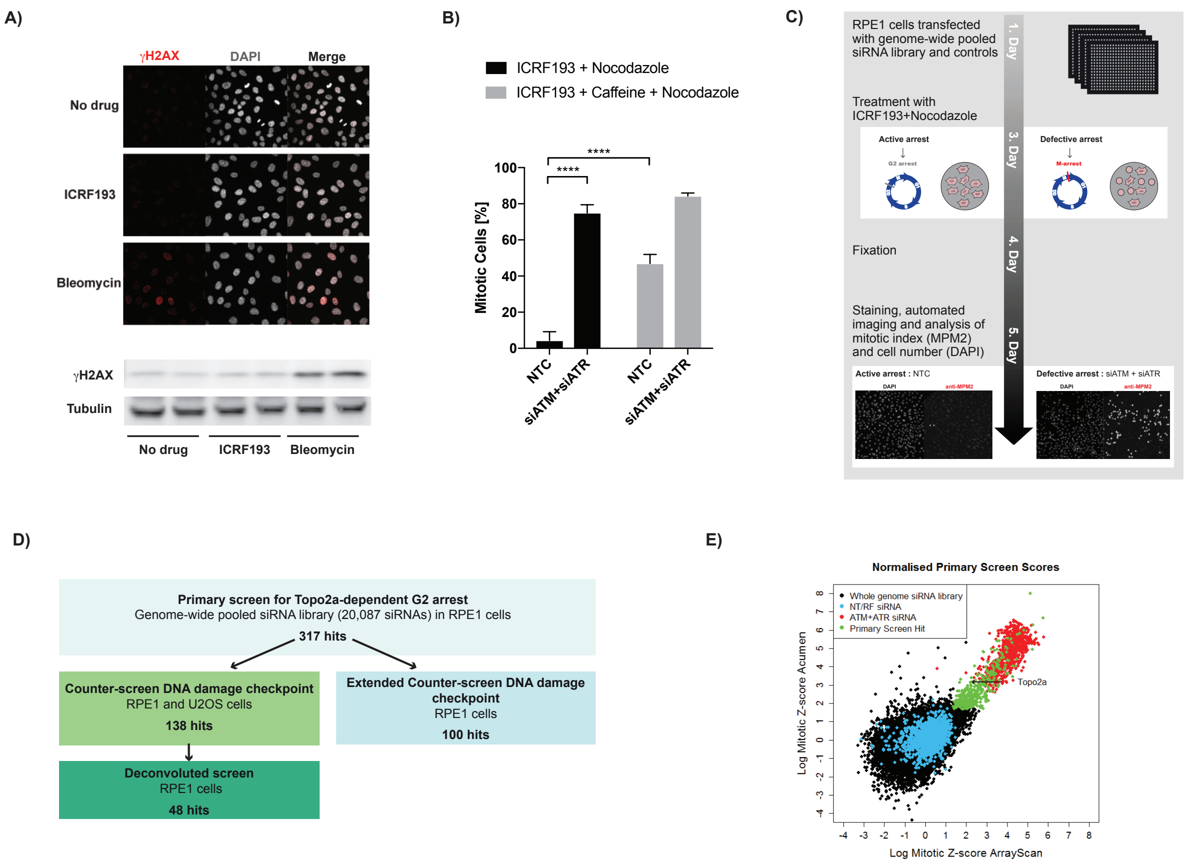

Fig S2: The Topo2a-dependent G2 arrest is mechanistically distinct from the DNA damage checkpoint

A) B) C)

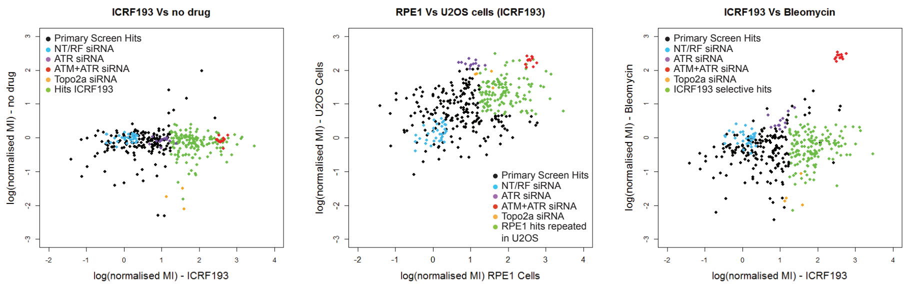

D)

No. of active siRNAs	No. of pools	Targeted genes
0/4	6 (4.3%)	s. Table S3
1/4	88 (60.6%)	s. Table S3
2/4	29 (21.2%)	ATP6V1F, C9ORF140, COG7, COL27A1, CSNK2B, DKFZP434I116, DPP7, FLJ14639, FLJ22173, FLJ38984, IDH1, IPO13, KIAA1914, LCE1C, LOC388722, TMEM81, LRP11, MGC10992, NUDT14, NUTF2, NXT1, OTOF, PGBD3, RLBP1, SLC35E1, SMC5, SP8, UNC5C, WFDC5
3/4	16 (11.7%)	BHLHB5, CDKN1A, COPS4, KIAA0853, MBD4, NSMCE1, NSMCE2, NSMCE3, P29, PD2, PDGFB, SLC6A20, SNAP29, THOC2, TOP2A, USP39
4/4	3 (2.2%)	SAE1, SMC6, PHF5A

E)

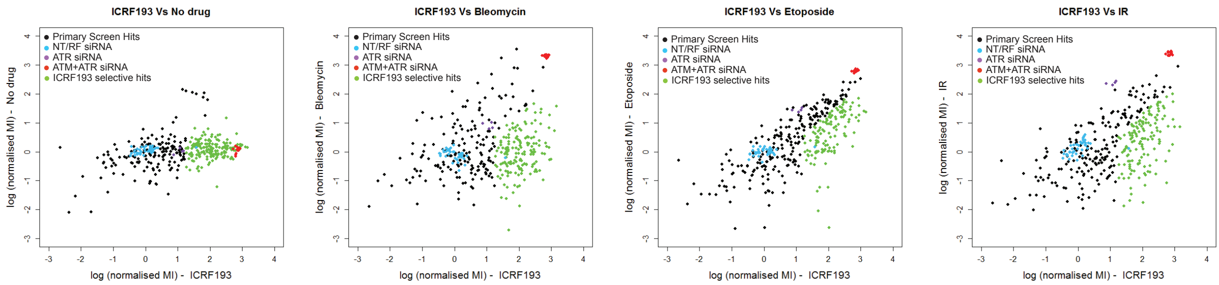

F)

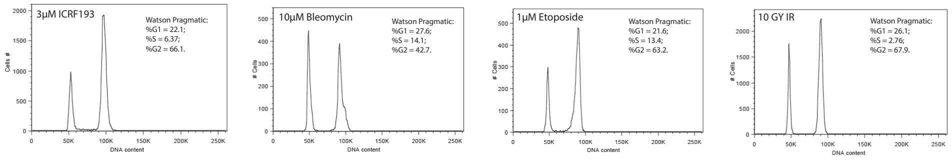

G)

	Number of input genes			
	48	58	68	78
**Nodes**				
**SMC5/6**	12	16	17	18
**THOC**	4	4	4	5
**PDGFB**	3	3	3	6
**ZC3H13**	2	2	3	3
**CDKN1A**	1	2	2	2
**COPS4**	1	1	1	2
**Edges**				
**SMC5/6**	24	32	34	35
**THOC**	3	3	3	6
**PDGFB**	2	2	2	7
**ZC3H13**	1	1	2	2
**CDKN1A**	1	1	1	1
**COPS4**	0	0	0	1

Fig S3: Validation that the SMC5/6 complex is required for the Topo2a-dependent G2 arrest

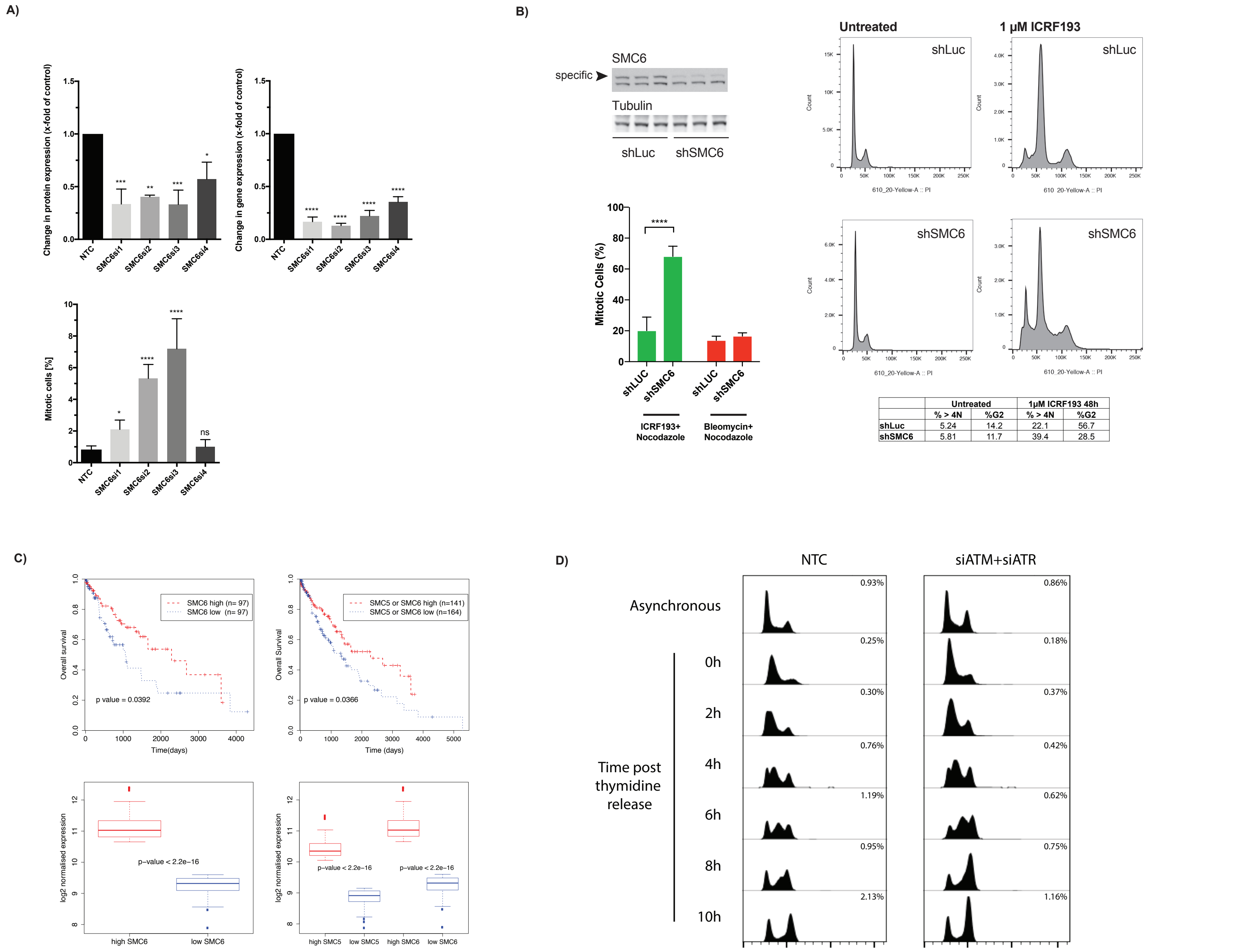

	Untreated		1µM ICRF193 48h	
	% > 4N	%G2	% > 4N	%G2
shLuc	5.24	14.2	22.1	56.7
shSMC6	5.81	11.7	39.4	28.5

Fig S4: SMC6 interacts with Topo2a

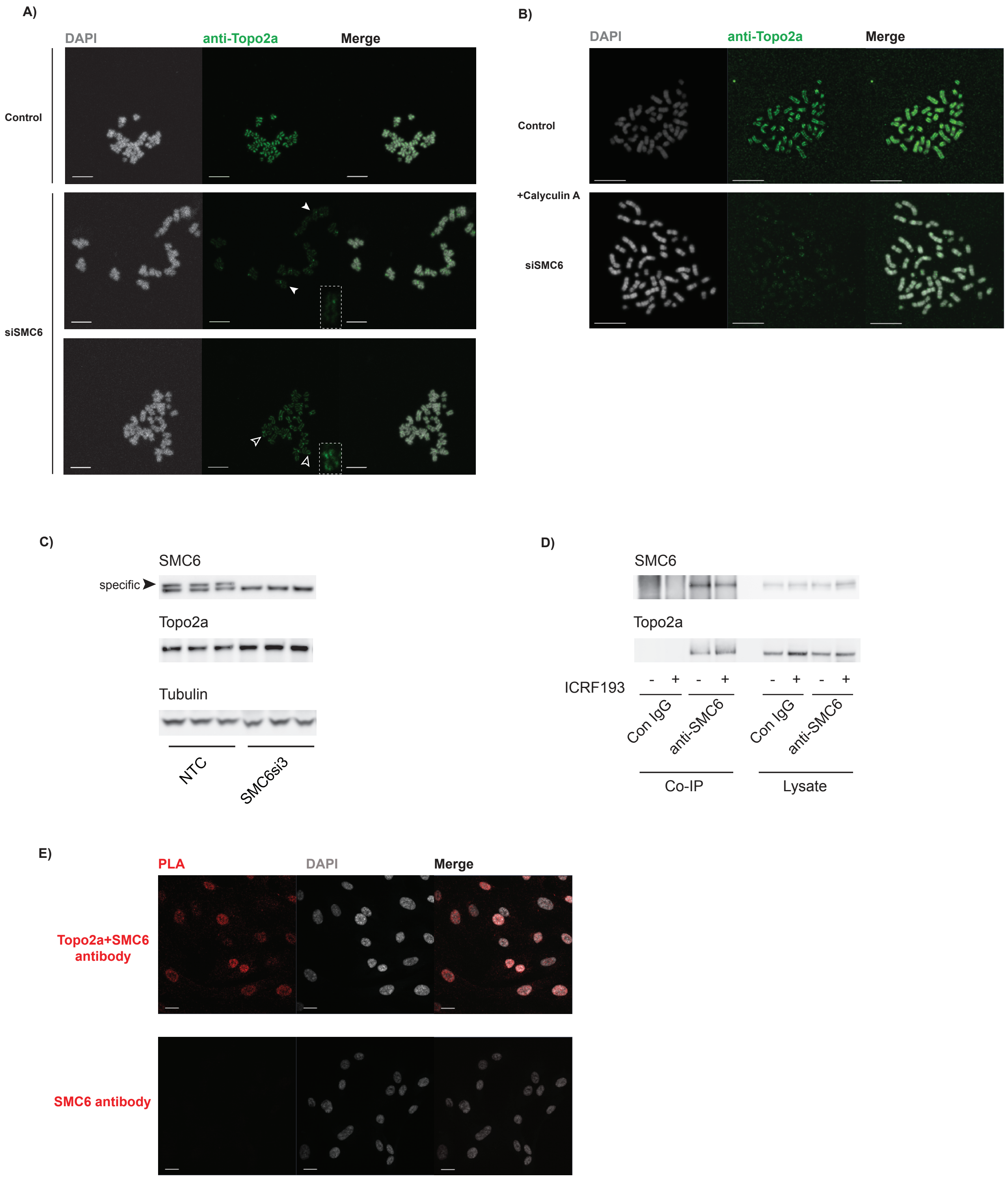

Fig S5: NSE2 SUMO ligase activity is required for chromosome segregation and arrest implementation

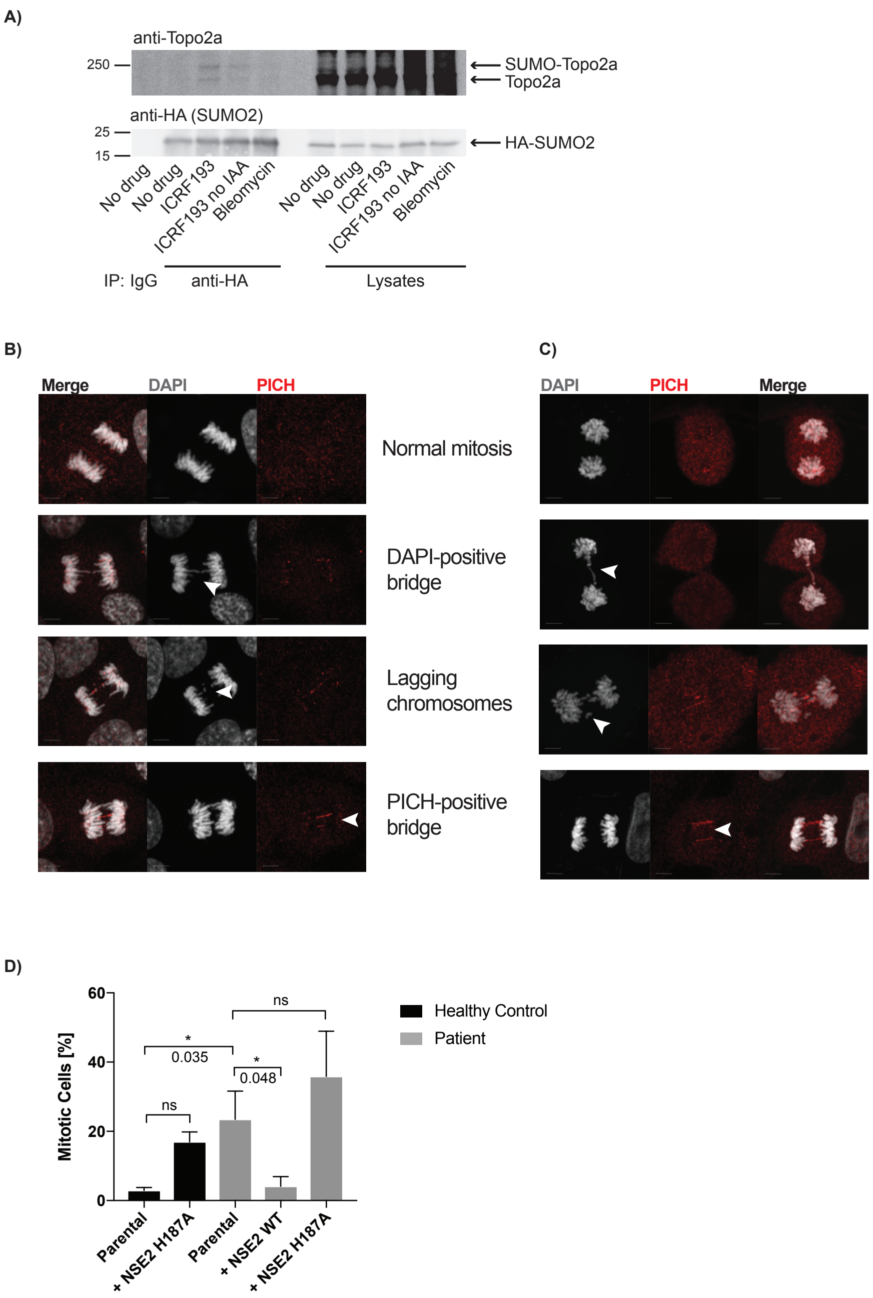

Fig S6: Topo2a is specifically SUMOylated by NSE2

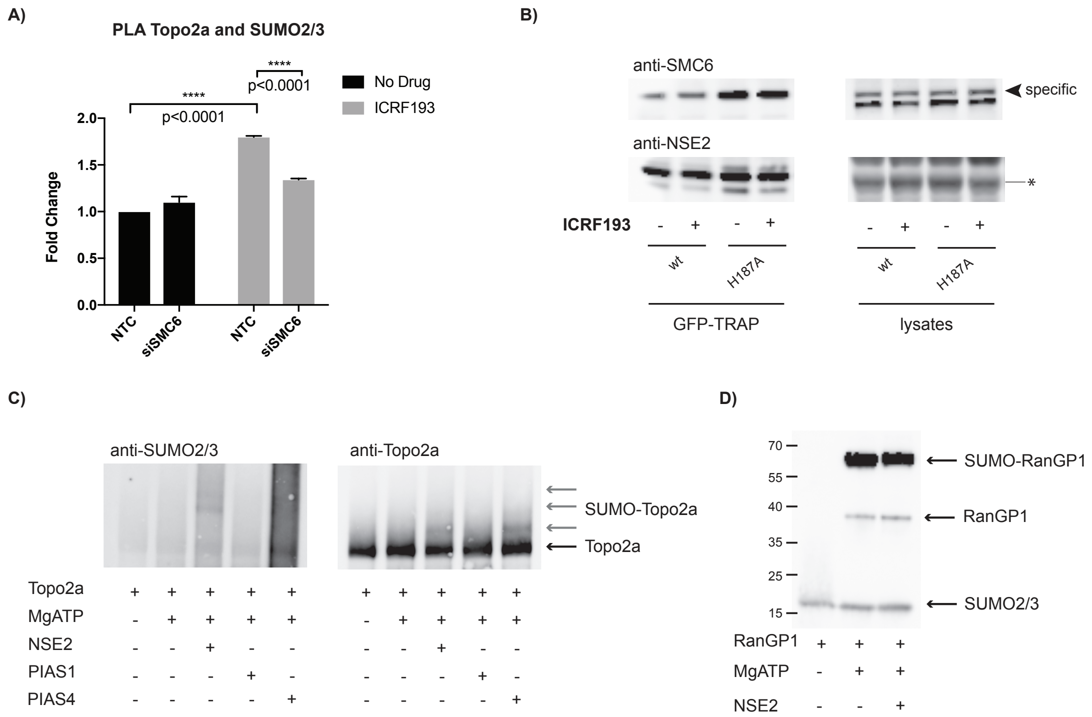

Fig S7: ICRF193 induces SUMOylation of Topo2a at the C-terminus

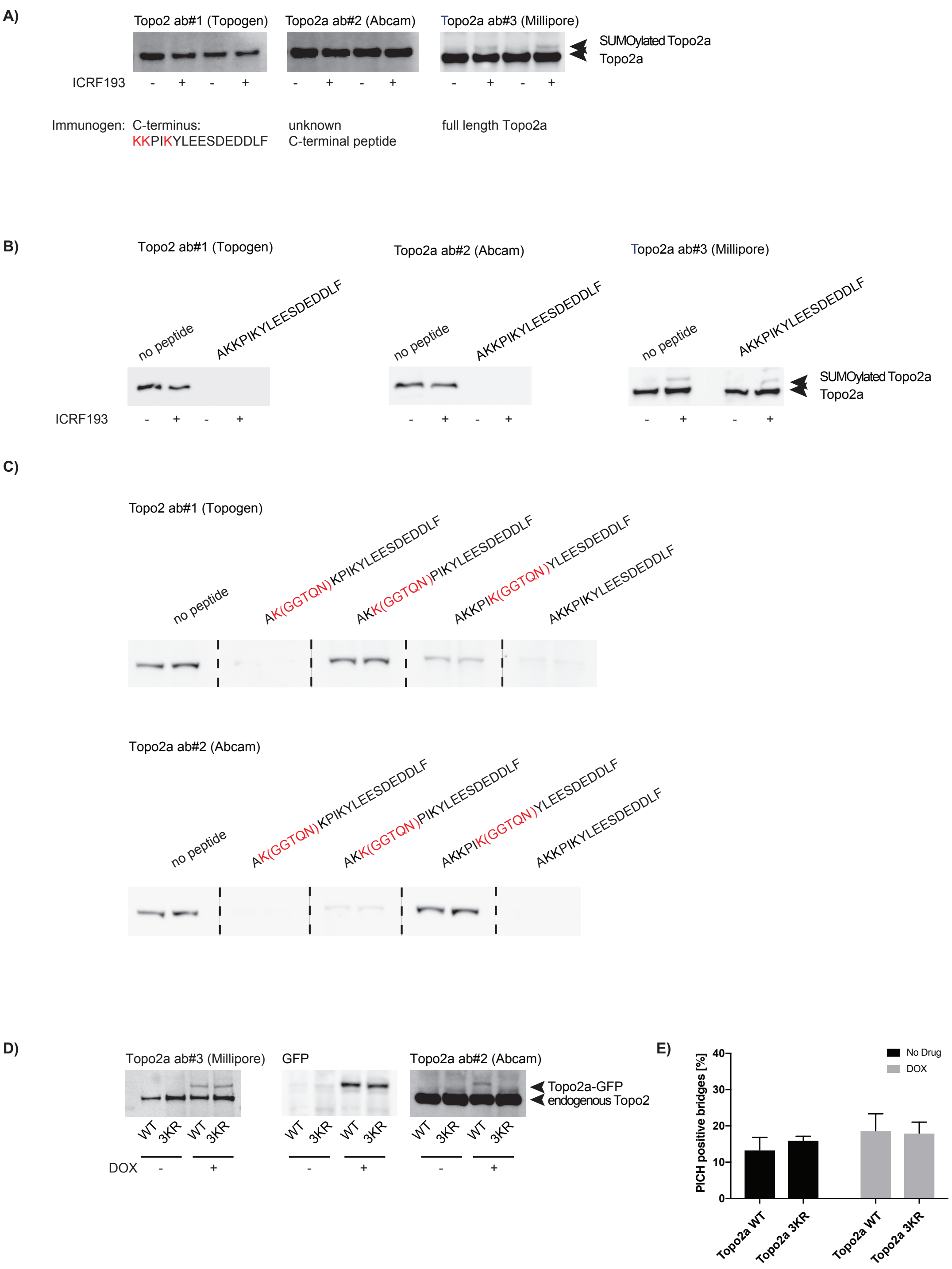

Supplement: Supplementary Data [file gky1295_supplemental_files.zip › SupplementaryFiguresandlegends_15122018.pdf]
